# Endoplasmic reticulum stress as a key mechanism in stunted growth of seawater rainbow trout (*Oncorhynchus mykiss*)

**DOI:** 10.1186/s12864-021-08153-5

**Published:** 2021-11-16

**Authors:** Bernat Morro, Richard Broughton, Pablo Balseiro, Sigurd O. Handeland, Simon Mackenzie, Mary K. Doherty, Phillip D. Whitfield, Munetaka Shimizu, Marnix Gorissen, Harald Sveier, Amaya Albalat

**Affiliations:** 1 Institute of Aquaculture, University of Stirling, Stirling, UK; 2 NORCE AS, Bergen, Norway; 3 Department of Biological Sciences, University of Bergen, Bergen, Norway; 4 Institute of Health Research and Innovation, Centre for Health Science, University of the Highlands and Islands, Scotland, UK; 5 Institute of Infection, Immunity and Inflammation, University of Glasgow, Scotland, UK; 6 Faculty of Fisheries Sciences, Hokkaido University, Sapporo, Japan; 7 Department of Animal Ecology and Physiology, Radboud University, Institute of Water and Wetland Research, Nijmegen, The Netherlands; 8 Lerøy Seafood Group ASA, Bergen, Norway

**Keywords:** Aquaculture, Insulin-like growth factor-I, Reactive oxygen species, Runt, Pathology, Oxidative stress, Salmonid, Smoltification, Steelhead

## Abstract

**Background:**

Rainbow trout (*Oncorhynchus mykiss*) is a salmonid species with a complex life-history. Wild populations are naturally divided into freshwater residents and sea-run migrants. Migrants undergo an energy-demanding adaptation for life in seawater, known as smoltification, while freshwater residents display these changes in an attenuated magnitude and rate. Despite this, in seawater rainbow trout farming all fish are transferred to seawater. Under these circumstances, weeks after seawater transfer, a significant portion of the fish die (around 10%) or experience growth stunting (GS; around 10%), which represents an important profitability and welfare issue. The underlying causes leading to GS in seawater-transferred rainbow trout remain unknown. In this study, we aimed at characterising the GS phenotype in seawater-transferred rainbow trout using untargeted and targeted approaches. To this end, the liver proteome (LC-MS/MS) and lipidome (LC-MS) of GS and fast-growing phenotypes were profiled to identify molecules and processes that are characteristic of the GS phenotype. Moreover, the transcription, abundance or activity of key proteins and hormones related to osmoregulation (Gill Na+, K + –ATPase activity), growth (plasma IGF-I, and liver *igf1*, *igfbp1b*, *ghr1* and *ctsl*) and stress (plasma cortisol) were measured using targeted approaches.

**Results:**

No differences in Gill Na+, K + –ATPase activity and plasma cortisol were detected between the two groups. However, a significant downregulation in plasma IGF-I and liver *igf1* transcription pointed at this growth factor as an important pathomechanism for GS. Changes in the liver proteome revealed reactive-oxygen-species-mediated endoplasmic reticulum stress as a key mechanism underlying the GS phenotype. From the lipidomic analysis, key observations include a reduction in triacylglycerols and elevated amounts of cardiolipins, a characteristic lipid class associated with oxidative stress, in GS phenotype.

**Conclusion:**

While the triggers to the activation of endoplasmic reticulum stress are still unknown, data from this study point towards a nutritional deficiency as an underlying driver of this phenotype.

**Supplementary Information:**

The online version contains supplementary material available at 10.1186/s12864-021-08153-5.

## Background

Industrialised intensive aquaculture is a more recent form of animal production than stockbreeding of terrestrial animals (i.e. poultry, cattle and pigs) and, by comparison, has had relatively few years to selectively breed the most desirable attributes [1]. Fish breeding programs rely heavily on advances in reproductive and molecular techniques to accelerate the selection of desirable traits [2]. The relatively low degree of domestication, particularly in some aquatic species, provides scientists with the opportunity to study unwanted commercial phenotypes that may not be present in the future. With the identification of the cause(s) of such phenotypes, their development could be prevented. For production species, fast growth is one of the main desirable attributes. Slow or stunted growth, on the other hand, is a phenotype that delivers negative impacts on production profitability, sustainability, and more importantly on animal welfare [3].

Rainbow trout (*Oncorhynchus mykiss*) is a salmonid species with a unique life-history. Wild populations are naturally divided into freshwater residents, which do not migrate to seawater, and sea-run migrants. The latter undergo an energy-demanding rheostatic process that prepares the organism for life in seawater while still in freshwater (i.e. smoltification), while freshwater residents display an attenuated magnitude and rate of these changes [4, 5]. The drivers towards either life history are largely unknown. Efforts to produce a strain with a single phenotype, either selecting the sea-run [4, 6, 7] or the freshwater-resident [8], could only increase the proportion of the desired phenotype. Production of seawater transferred-rainbow trout is still relatively small but both investment and production have increased steadily in recent years [9]. The animals grown in these production systems are only a few generations away from freshwater-farmed rainbow trout, which were selected for their performance in freshwater systems. Under these circumstances, a persistent problem has affected this sector: shortly after seawater transfer, a portion of the animals (around 10%) die, and another portion (around 10%) experience reduced growth, a marked decrease in condition factor, and poor welfare [10, 11]. These animals are known as growth-stunted (GS) or runts (in Norwegian *pinne*, meaning stick).

Few published studies have investigated the GS seawater-transferred rainbow trout phenotype. Two studies found nematode infections in a small proportion of the GS rainbow trout: Roiha et al. [12] detected it in 5 runts out of 178 discarded fish and not in harvest quality fish, and Skov et al. [13] detected it in 9.5% of the runts and 2.1% of the harvest quality fish. This was likely due to the reported feeding habits of the GS fish, which consumed more small fish, crustaceans and biofouling species than their healthier counterparts [13], and not an underlying cause of GS development. Previous studies identified a freshwater rainbow trout phenotype, as also seen in other salmonid species [14–16], that occurs in both aquaculture and laboratory conditions and presents stunted growth and reduced condition factor [17, 18]. Studies have shown that while reduced food intake is an important factor for the development of this phenotype in rainbow trout [18, 19], fundamental physiological traits such as a higher standard metabolic rate are key intrinsic drivers in brown trout (*Salmo trutta*) [15]. Other studies have linked GS to subordinate behaviour, with subordinate fish exhibiting higher post-stress plasma cortisol levels and lower stressor avoidance [20, 21]. From a metabolic perspective, subordinate fish rely on β-oxidation of circulating free fatty acids rather than on triacylglycerides (TAGs) for energy [22]. Moreover, differences in carbohydrate metabolism in subordinate fish include a lower hepatic glycogen content, higher gluconeogenic potential [23–25] and higher plasma glucose levels [18, 26, 27]. From a protein perspective, results so far have been inconclusive with no significant changes reported in liver protein levels, with only moderate, but not significantly different, aspartate aminotransferase and alanine aminotransferase activities detected [18]. These listed mechanisms may be relevant to the GS phenotype in seawater-transferred rainbow trout but, interestingly, the problem at hand occurs only after an artificial and forceful seawater transfer of the fish.

Several explanations, such as increased stress, subordinate social status, incomplete smoltification or fasting are possible causes. In cases like this, the initial characterisation of the phenotype can be the way forward, providing a holistic view of the underlying mechanisms that lead to a condition [28–30]. This is where -omics technologies exceed, measuring the abundance of large numbers of posteriorly annotated biomolecules (i.e. transcripts, proteins, metabolites, lipids, etc.) of a sample in an untargeted manner. With this information, it is possible to pinpoint the involved pathways before moving onto a targeted approach to study them in detail. In similar cases within both humans and other animal species, −omics approaches have been successfully used, with both proteomic and lipidomic studies having been used to assess the effects on liver tissue of differential feeding [31–33], stress [34], and disease [35, 36], among others.

As a target tissue, the liver, due to its central role in energy storage and mobilization, is ideal as it should reflect differences in energy balance and metabolism [37]. Liver pathologies like hepatic steatosis (i.e. fatty liver) have been linked to loss of appetite and dwarfism in animals like cattle and horses [38, 39], which in turn have been attributed to endoplasmic reticulum (ER) stress [40, 41]. The ER is central to the processes of energy production and protein synthesis. ER stress prevents the correct function of the organelle and it is intrinsically linked with the dysregulation of lipid metabolism [41]. One of the main consequences of ER stress is an accumulation of mis-folded proteins in the ER lumen and impaired protein glycosylation. Folding depends on the correct function of lectins like calreticulin and calnexin [42], while other proteins like Heat Shock Protein 90 (HSP90) stabilize the system [43]. ER stress is closely related to oxidative stress and redox homeostasis [44]. ER stress in hepatic disorders like steatosis have been linked to abnormally regulated proteins such as glycine N-methyltransferase [45], catalase [46], and alpha-2-macroglobulin [47]. Pathogens can also drive ER stress. For instance, the salmonid pathogens *Francisella* spp. have been shown to disrupt the transcription of enzymes involved in glycosylation or deglycosylation [48]. Currently, there is no data to support whether the GS phenotype is linked to oxidative and ER stress although these processes have been linked to several metabolic disorders that can negatively affect health and growth [49, 50].

The aim of this study was to characterise the GS phenotype using targeted and untargeted approaches. To this end, the liver proteome (liquid chromatography - tandem mass spectrometry (LC-MS/MS)) and lipidome (liquid chromatography - mass spectrometry (LC-MS)) of GS and FG phenotypes were profiled to pinpoint molecules and processes that are characteristic of the GS phenotype. Moreover, the transcription of several genes (*insulin-like growth factor 1 (igf1*), *insulin-like growth factor binding protein 1b (igfbp1b*), *growth hormone receptor 1 (ghr1*) and *cathepsin L* (*ctsl*), as well as abundance (plasma IGF-I and cortisol) and activity (Gill Na^+^, K^+^–ATPase (NKA)) of key proteins and hormones related to osmoregulation (NKA activity), growth (plasma IGF-I, *igf1*, *igfbp1b*, *ghr1* and *ctsl*) and stress (plasma cortisol) were analysed using targeted approaches.

## Results

### Seawater tolerance in FG and GS fish

**Fig. 1:**
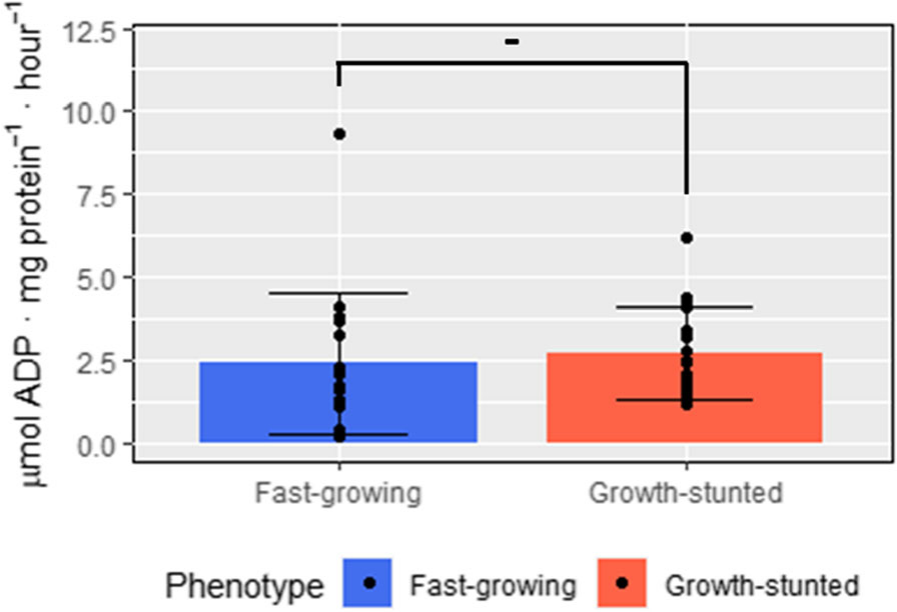
Gill Na+, K + –ATPase activity in FG and GS fish**.** Samples were taken after 9 weeks in seawater (*n* = 17)

NKA activity values after 9 weeks in seawater were not related to GS development as no significant differences among FG and GS groups (*p* = 0.60, df = 32, t = 0.53) were observed. Values for both groups were 2.6 ± 0.30 μmol ADP mg protein^− 1^ h^− 1^ on average (Fig. 1).

### Plasma cortisol in FG versus GS fish

**Fig. 2:**
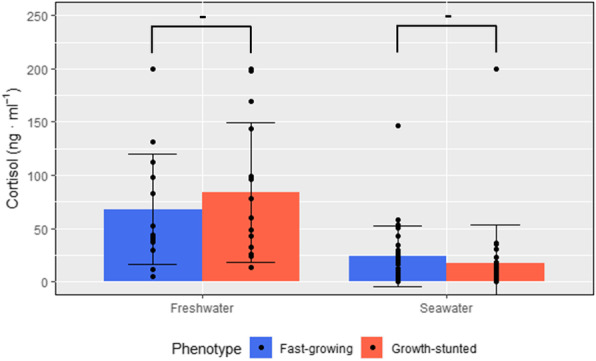
Plasma cortisol levels in FG and GS fish. Samples were taken prior (left, *n* = 15) and 9 weeks after (right, *n* = 30) seawater transfer

Plasma cortisol levels showed no significant differences among phenotypes in either freshwater (*p* = 0.47, df = 28, t = 0.73) or seawater (*p* = 0.44, df = 58, t = 0.78). However, cortisol levels were significantly higher when fish were in freshwater (average of 76.0 ± 10.67 ng ml^− 1^) compared to seawater (average of 20.8 ± 4.19 ng ml^− 1^) (*p* < 0.0001, df = 88, t = 5.76) (Fig. 2).

### Growth-related parameters in FG versus GS fish

**Fig. 3:**
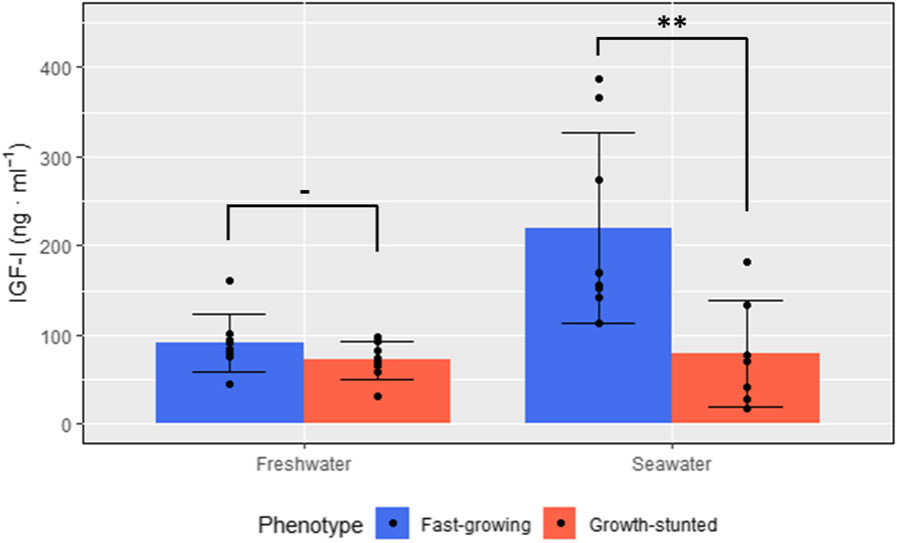
Circulating IGF-I abundance in FG and GS fish. Samples were taken prior (left) and 9 weeks after (right) seawater transfer (*n* = 8)

Circulating plasma IGF-I levels showed no significant differences among phenotypes in freshwater (*p* = 0.18, df = 14, t = 1.46) but did after 9 weeks in seawater (*p* < 0.01, df = 13, t = 3.09) (Fig. 3). This significant difference was due to a significant increase for FG (p < 0.01, df = 7, t = 3.76), while plasma IGF-I levels did not vary significantly from freshwater to seawater for GS (*p* = 0.63, df = 7, t = 0.50).

**Fig. 4:**
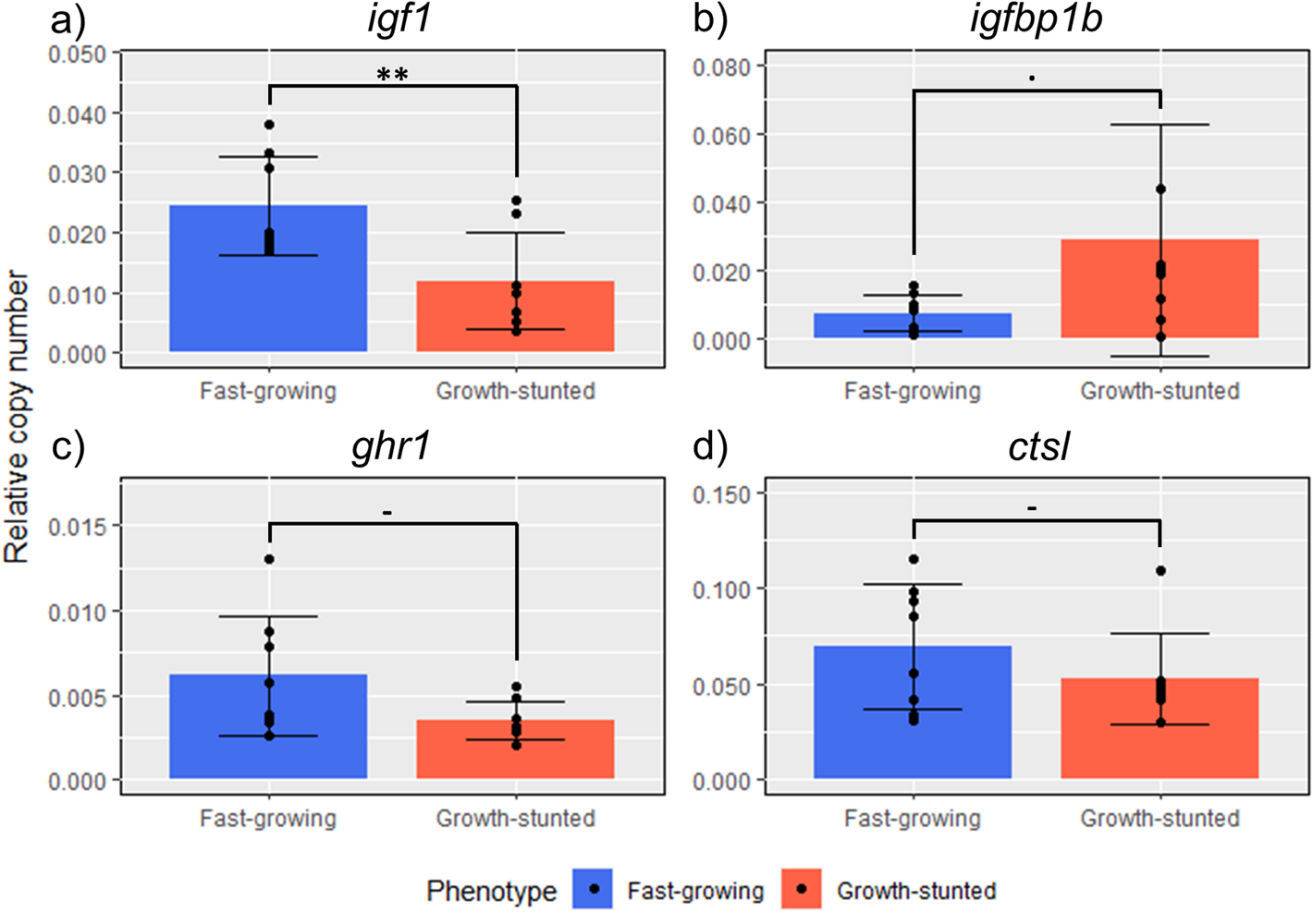
Liver transcription of growth-related genes. Genes *igf1* (a), *igfbp1b* (b), *ghr1* (c), and *ctsl* (d) in FG and GS fish after 9 weeks in seawater (*n* = 8)

After 9 weeks in seawater, concordantly with plasma IGF-I abundance, liver *igf1* transcription was higher in FG than in GS (*p* < 0.01, df = 14, t = 3.99). On the other hand, while not significant for *igfbp1b*, there was weak evidence (i.e. 0.05 < *p*-value < 0.1) indicating that its transcription might be higher in GS (*p* = 0.07, df = 14, t = 1.94). For *ghr1* (*p* = 0.10, df = 14, t = 1.73) and *ctsl* transcription (*p* = 0.39, df = 14, t = 0.90), no differences were observed (Fig. 4).

### Liver proteome analysis

The protein fraction in the liver of FG fish was significantly lower (14.6% ± 0.75) than in GS fish (17.5% ± 1.05) (*p* < 0.01, df = 8, t = 4.94). After MS analysis of TMT labelled liver samples, a total of 540 peptides were detected (Additional file 1). Of those, 299 peptides were uniquely assigned to proteins and used for quantification. These peptides were mapped onto 132 quantified proteins.

**Fig. 5:**
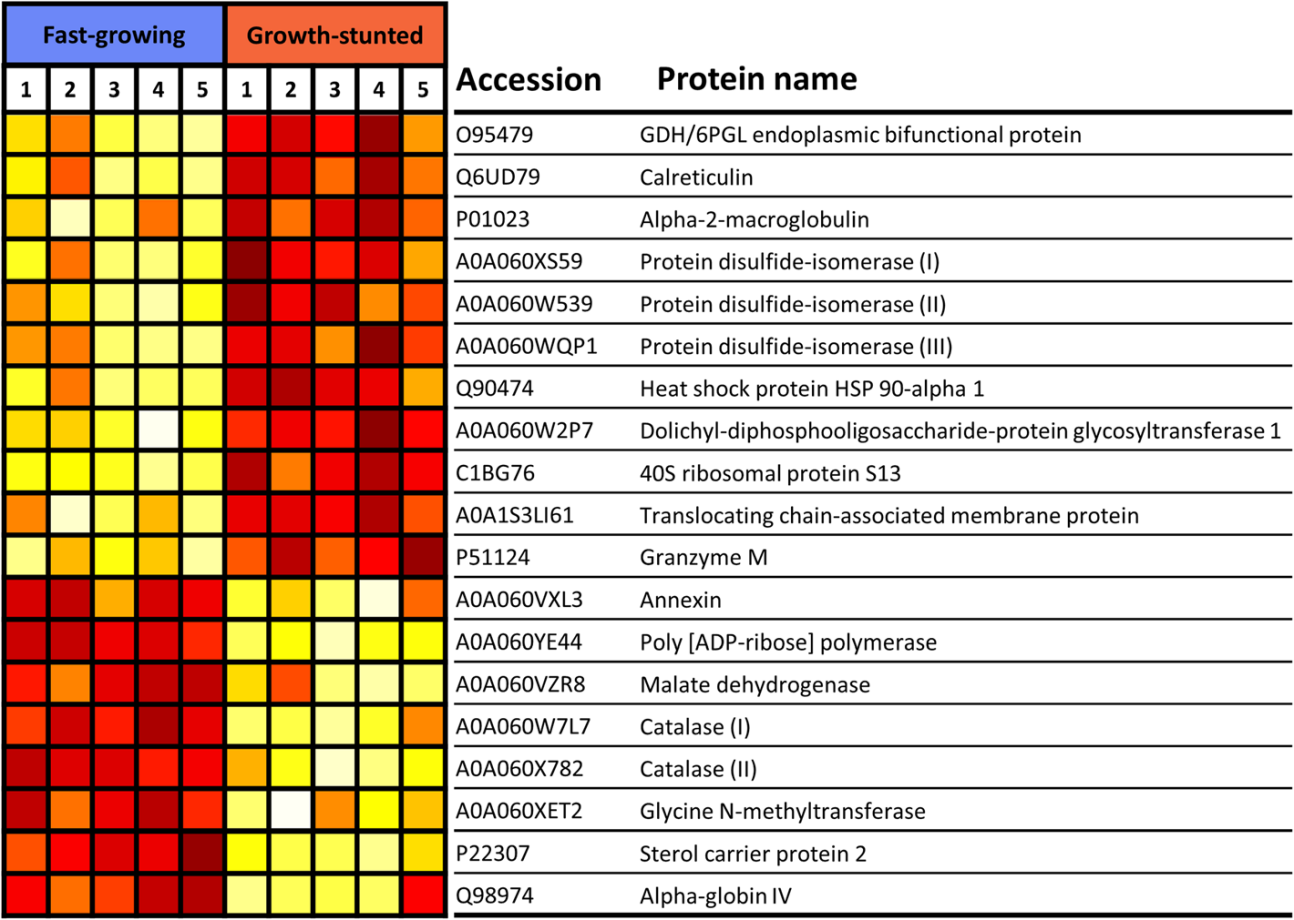
Heatmap of protein abundance for 19 differential proteins between FG and GS liver samples (*n* = 5). Accession and protein name are retrieved from Uniprot (uniprot.org). Colour goes from white (less abundant) to dark red (more abundant) and each row is scaled independently

In total, 19 differential proteins (q-value < 0.05) were detected when comparing the liver proteome of GS and FG (Fig. 5). These proteins were assigned functionally to translation, redox homeostasis, oxygen transport, stress response, and transport and metabolism of carbohydrates and lipids. Among upregulated differential proteins in the GS phenotype there were a number of critical protein chaperones previously reported to be associated with reactive oxygen species (ROS) mediated ER stress: calreticulin, protein disulphide isomerases (PDI; I, II and III), HSP90-α1, alpha-2-macroglobulin. Other proteins associated with the ER and involved in the translocation of secretory proteins (translocating chain-associated membrane protein) or involved in providing reducing equivalents to maintain adequate levels of reductive cofactors in the ER (GDH/6PGL endoplasmic bifunctional protein) were also upregulated. On the other hand, downregulated differential proteins in the GS phenotype included catalase (I and II), a crucial antioxidant enzyme, the inhibition of which has been reported under ER stress conditions, and other proteins also reported to be closely linked to ER stress and redox homeostasis such as Glycine N-methyltransferase or involved in lipid and energy homeostasis, such as annexin, sterol carrier protein 2 (SCP-2) and malate dehydrogenase.

### Liver lipidome analysis

**Fig. 6:**
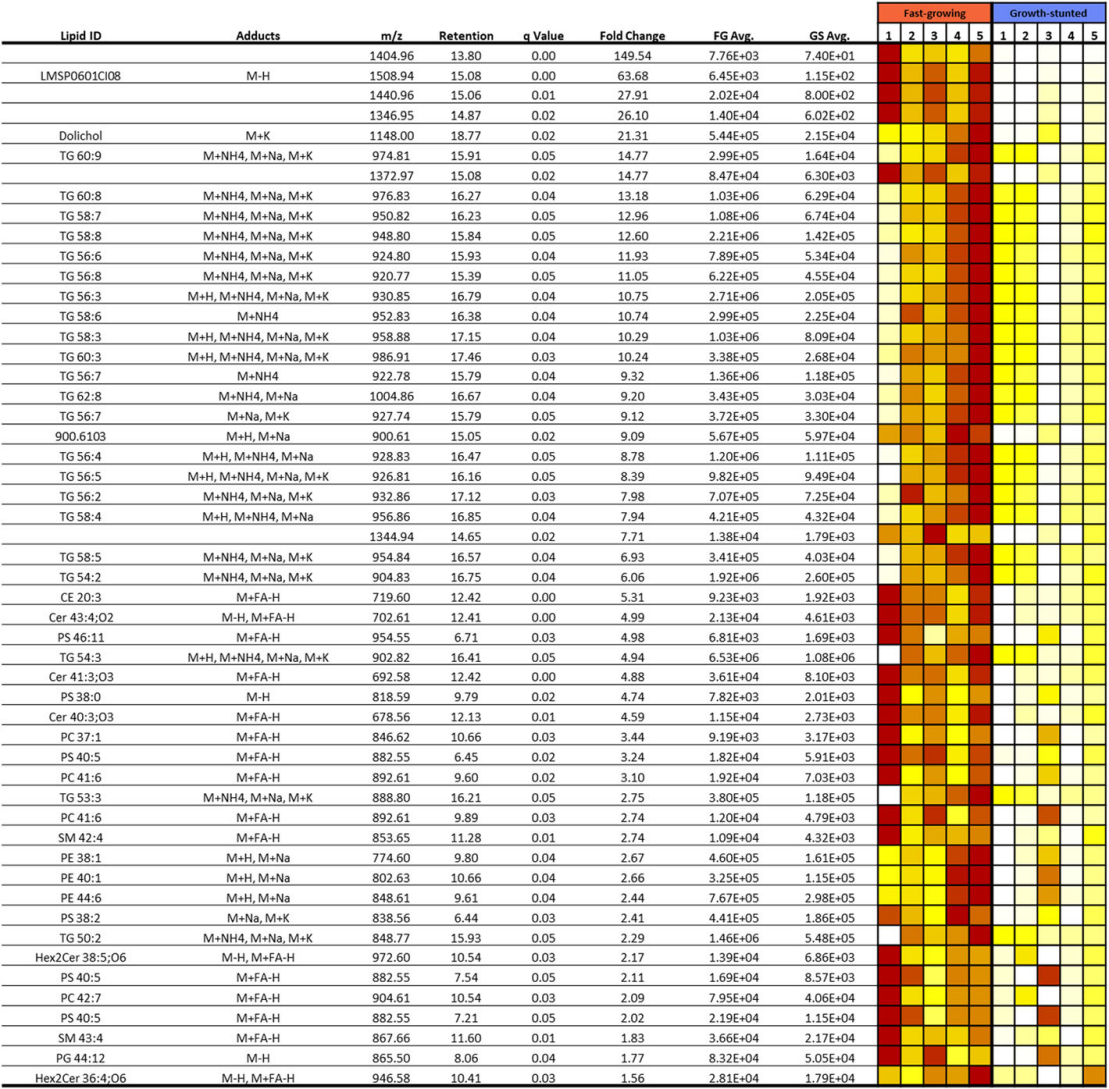
Significantly higher lipids in the fast-growing phenotype (*n* = 5). GS: Growth-stunted, FG: Fast-growing. Abundance values are based on normalised ion counts. Colour goes from white (less abundant) to dark red (more abundant) and each row is scaled independently. Retention is expressed in minutes

Lipid levels in the liver of FG fish were not significantly different (15.4 ± 2.87%) to levels in GS fish (13.8 ± 0.97%) (*p* = 0.60, df = 8, t = 0.54). Lipidomics analysis of liver samples returned 6456 and 2756 lipid features in positive and negative modes, respectively. OPLS-DA analysis (Suppl. Fig. 1, Additional File 2) revealed that neutral lipids, specifically TAGs, as well as ceramides and a cholesteryl ester were found to be the main drivers in separating the FG fish from the GS fish. TAGs ranged from 52 to 60 carbon moieties, however the 56–60 carbon TAGs appeared to dominate, with a wide range of unsaturation levels, again ranging from 1 to 9 double bonds. With regards to peak abundance, TAG peak areas were found to be highest with peak areas in the 10^6^ and 10^7^ ion count range. However, care must be taken with regard to inter-lipid comparisons, as a fold change quantitation method was used, which is not amenable to absolute lipid quantitation. Six unknown features (unnamed Lipid ID rows plus LMS0601CI08 in Fig. 6) were also detected which exhibited large fold changes, albeit with much lower abundances (Fig. 6, Additional file 2).

In general, the phospholipids which distinguish the GS condition tended to be larger at 44 carbons in length, as well as being more unsaturated, containing on average 10 double bonds. There was also some compound identity ambiguity, based on mass accuracy alone, which yielded either phosphatidyl serine or inositol-based sphingolipids, with these compounds generally being more abundant in the FG fish.

**Fig. 7:**
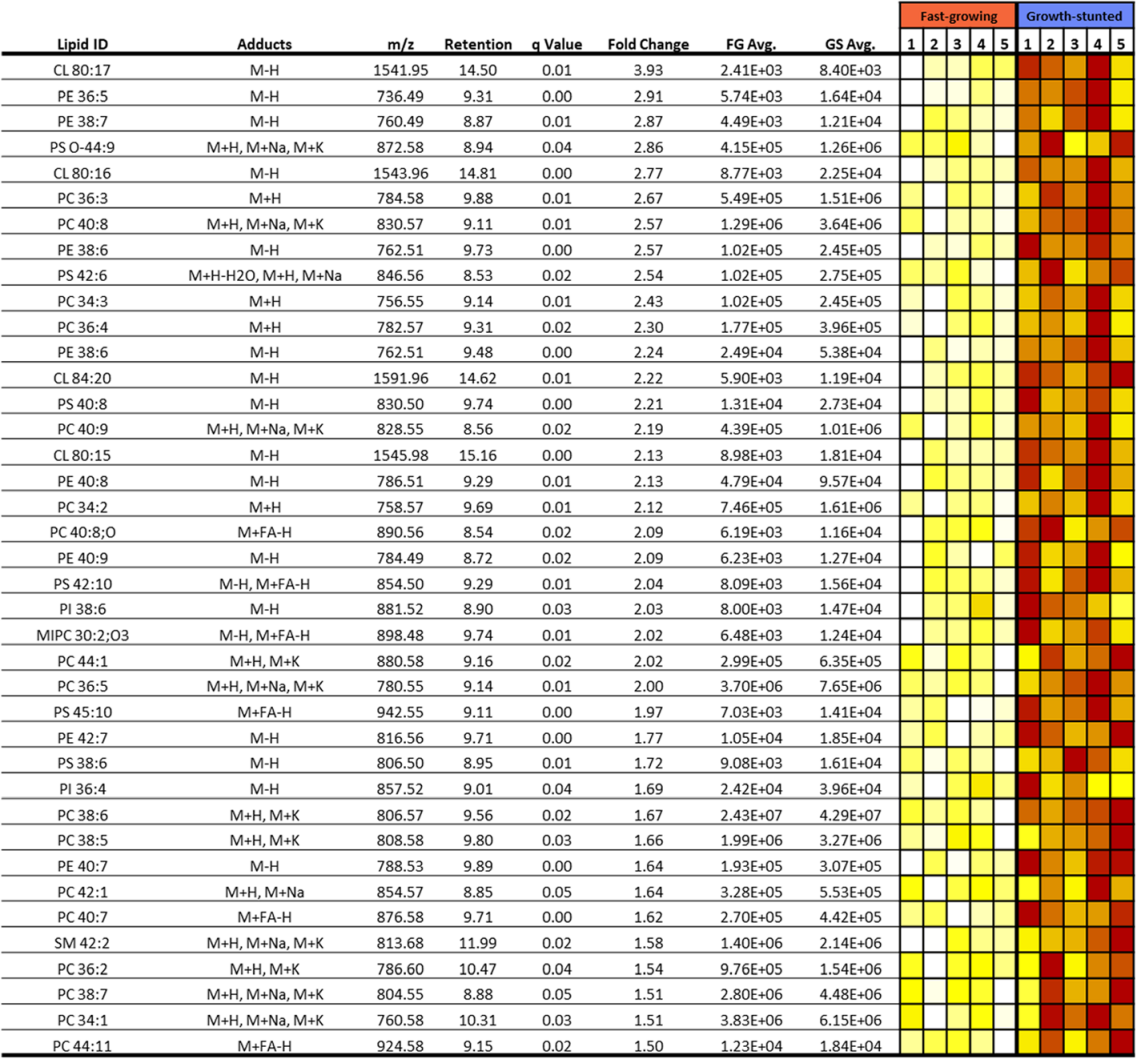
Significantly higher lipids in the growth-stunted phenotype (n = 5). GS: Growth-stunted, FG: Fast-growing. Abundance values are based on normalised ion counts. Colour goes from white (less abundant) to dark red (more abundant) and each row is scaled independently. Retention is expressed in minutes

In total, 55 differential lipids were significantly higher in FG (fold change > 1.5; q-value < 0.05) compared to GS. The most significant changes in terms of numbers (24) and the greatest fold changes (ranging from 2.8 to 14.8) were observed in TAG, with most TAGs experiencing a fold change greater than 5 in the FG fish (Fig. 6). On the other hand, in GS liver, 39 lipids were significantly more abundant (fold change <− 1.5; q-value < 0.05), all of which were phospholipids; phosphatidylcholine (PC; 15), phosphatidylethanolamine (PE; 7), phostidylserine (PS; 4) and cardiolipins (CL, 4) (Fig. 7). In fact, both conditions demonstrated fluctuations within the main classes of phospholipid, including PC, PE and PS, with PG 44:12 only detected in FG fish and phosphatidylinositol (PI) 38:6 and 36:4 only detected in GS fish. The phospholipids in general were elevated in the GS fish, as illustrated by the approximately 2.5-fold increase in the number of phospholipids. GS fish also produced elevated amounts of CL, which was a characteristic lipid class associated with this phenotype.

## Discussion

Seawater-transferred rainbow trout farming is a developing industry, which has experienced a strong increase in production during the last three decades [9]. However, the incidence of GS fish has hindered its economic growth and is an animal welfare concern. The cause of the development of this unwanted phenotype remains unknown. Understanding the underpinning metabolic traits that characterise this phenotype could be the first step towards optimized rearing strategies and feeding protocols that decrease the incidence of GS. To this end, untargeted –omics approaches were used to study the liver proteome and lipidome of GS fish compared to FG fish, while targeted approaches were employed to measure smoltification status (NKA activity), growth-related (plasma IGF-I abundance and liver *igf1*, *igfbp1b*, *ghr*, and *ctsl* transcription) and stress-related parameters (plasma cortisol).

Since growth is the attribute that most clearly distinguishes GS and FG fish, it would be expected that growth-related molecular drivers differ between the two phenotypes. As determined by a genome-wide association studies [51, 52], somatic growth is polygenic trait in rainbow trout. It is associated to single-nucleotide polymorphisms in several candidate genes including genes involved in development processes, growth factors, and bone tissue and nutrient metabolism. It is highly likely that the GS condition is also genetically determined, which would justify breeding programs to reduce the appearance of GS. Some genes and proteins are classically measured as determinants of growth. IGF-I is a peptide hormone that promotes growth in fish, as is IGF-II [53–55], along with growth hormone and its receptor GHr1, among others. Among the IGFBPs (1 to 6) present in rainbow trout, IGFBP1b was of especial interest as it inhibits the binding of IGF-I to its receptor and represses growth [56, 57]. Both IGFBP1b and CTSL, an endopeptidase, are induced under catabolic conditions [58, 59]. Indeed, differences between the two phenotypes were found in some of these growth-regulatory molecules. No evidence to confirm changes in the expression of *ghr1*, *igfbp1b* or *ctsl* was obtained, but the involvement of IGF-I was demonstrated. IGF-I was significantly lower in GS fish at both plasma and liver transcription levels. These results reiterate the importance of the somatotropic axis in the regulation of growth and show their involvement in the development of GS [60]. Moreover, while plasma IGF-I increased in FG fish during their time in seawater, levels in GS did not vary. This suggests that GS development might be associated to suboptimal seawater adaptation, which may trigger the development of this phenotype after animals are forcefully transferred to seawater. Another hypothesis for the reduction in growth once fish are transferred could be that GS fish become subordinate fish. Other studies have demonstrated how subordinate social status has a detrimental effect on growth, which is accompanied by a metabolic mobilisation of reserves [18, 61]. However, in such cases, metabolic changes have been, at least in part, driven by chronically high levels of plasma cortisol in the subordinate fish [17, 18, 62]. In the present study, plasma cortisol levels were similar between both groups suggesting that this phenotype is unlikely to be triggered by the development of a subordinate social status when animals are transferred at sea. Furthermore, from a metabolic perspective, subordinate fish have been shown to rely on β-oxidation of circulating free fatty acids rather than on triglycerides for energy [22] a feature not observed in the seawater-transferred GS phenotype.

The liver is a key organ for the accumulation and mobilization of energy reserves [37]. A labelled proteomic approach was used to unravel differences in the liver proteome between FG and GS. From a protein perspective, several proteins from the 19 identified differential proteins consistently pointed towards ER stress in GS livers. The ER is central to the processes of energy production, protein and lipid synthesis and it is closely related to oxidative stress and redox homeostasis [44]. In this study, an upregulation of chaperones known to increase in response to ER stress such as calreticulin and PDI [63, 64] was detected in GS livers. Downregulated differential proteins in the GS phenotype included catalase (I and II), a crucial antioxidant enzyme, the inhibition of which has been reported under ER stress conditions. Other proteins also reported to be closely linked to ER stress and redox homeostasis, such as glycine N-methyltransferase, or involved in lipid and energy homeostasis, such as annexin, SCP-2 and malate dehydrogenase were also differentially expressed in the two phenotypes [65, 66]. The increase, and more recently the proven relocation of stress induced ER cytosol chaperones to the cell plasma membrane have expanded the understanding of the functional role of these proteins, which can modulate immune responses in response to proteotoxic stress [67]. Within this context, ER stress has been shown to induce inflammation. The release of cytokines can be directly induced by ER/unfolded protein response (UPR) pathways or indirectly through the interaction with innate immune cells. In other organisms, the cross-talk of Toll-like receptors and ER/UPR pathways is associated to viral infections due to increased viral protein synthesis and assembly. This hypothesis cannot be discarded, as in the GS phenotype, besides the indication of ER stress, an increase in granzyme M was detected. This protein is a chymotrypsin-like serine protease that is abundantly expressed in innate effector natural killer cells and acts as a first line of defence against virus-infected or transformed tumour cells [68]. With current data, it is not possible to map out if the activation of ER/UPR pathways is a trigger or a consequence of an inflammatory state, a question that has been previously raised [69].

Other proteins upregulated in GS livers included the chaperone HSP90-α1, alpha-2-macroglobulin (broad spectrum protease inhibitor) and other proteins also associated with the ER. These proteins are involved in the translocation of secretory proteins (i.e. translocating chain-associated membrane protein) or involved in providing reducing equivalents to maintain adequate levels of reductive cofactors in the ER (i.e. GDH/6PGL endoplasmic bifunctional protein) [70, 71]. Therefore, our proteomic results in GS point towards ER stress as a key mechanism reflecting a state of functional imbalance.

As already mentioned, the ER is a key organelle of cellular lipid synthesis coordinating the transfer of lipids at the cellular level with ER stress associated with anomalous lipid metabolism [72]. In this respect, ER stressors can disrupt lipid metabolism. Lipidomic data showed important differences in the hepatic lipid composition of the two phenotypes. The biggest differences between FG and GS livers were found in energy reserve species (i.e. TAGs), which were lower in GS fish livers. Other studies have shown how ER stressors such as starvation and nutrient deficiencies can modulate autophagy, which plays a vital role in cell survival under long-term ER stress situations [73]. Autophagy provides cells with energy by mobilising energy stores such as TAGs. This mobilisation of lipid reserves is clearly observed in GS fish livers.

Other lipids which differentiated the two phenotypes were found in the phospholipid fraction. Phospholipids are key structural constituents of cellular membranes and of lipoproteins involved in the transport of dietary lipid from the intestine and liver to the rest of the body [74, 75]. In general terms, glycerophospholipids were elevated in GS fish livers, mainly PC, PE, PS and CL. Previous studies have reported that changes in PC species associated with dietary vegetable oil intake may cause an abnormal lipid deposition in the liver [76], which has also been linked to reduced growth [77] and suppressed immunity and antioxidant capacity [78]. Perturbations of glycerophospholipids, especially PC and PE levels, can result in lipid bilayer stress, which in turns causes ER stress [79]. Furthermore, being components of membranes, glycerophospholipids are affected by oxidative stress. This is especially the case for CL, due to their almost exclusive location in mitochondrial membranes where the electron-transport chain occurs, and where there is intense ROS production [80]. CL are involved in the biogenesis, dynamics and organization of mitochondrial membranes, controlling their permeability and contributing to the assembly of mitochondrial protein complexes involved in respiration and energy production [81, 82]. These lipids, which were elevated in GS fish livers, can be used as biomarkers for mitochondrial dysfunction [82, 83].

Some interesting associations between the proteomics and lipidomics datasets were also found. For instance, the higher abundance of the differential protein SCP-2 in the FG group is likely related to the higher abundance of TAGs found in this group. SCP-2 is thought to transfer cholesterol and phospholipids from the inner ER membrane to the plasma membrane [84], binding both fatty acids and isoprenoids such as dolichol [85], and facilitating the esterification of cholesterol to cholesteryl-esters. This putative SCP-2 was more abundant in FG fish, with C20:3 cholesteryl-ester also found to be elevated within the same condition, albeit at low absolute levels. The abundance of TAG in FG fish livers likely results in the formation of lipoproteins within the blood, resulting in the translocation of cholesterol and phospholipid to the plasma membrane, as well as the upregulation of cholesteryl-esters as a means of storage. Also of interest is the decreased abundance, 21-fold, of dolichol in GS fish livers. GS fish were found to have elevated dolichyl-disphophooligosaccharide-protein glycosyltransferase subunit 1 protein, which correlates with the reduction in the dolichol substrate pool. Decrease in dolichol has been proposed as a marker of aging, as well as of calorie restriction. In calorie restricted mice, dolichol was found to decrease in the liver [86], with this trend appearing to be present within the GS fish, this may indicate that at least to some extent, GS are experiencing caloric restriction.

CL are implicated in the energetic balance [81, 82] and ceramides regulate a wide variety of molecular processes [87, 88]. While they are very different in composition and nature, both lipid classes are prone to peroxidation, which can lead to dysfunctional mitochondria in the case of CL [80], and to the induction of apoptosis for ceramides [87, 89]. In this sense, the differential proteins catalase (I and II) and PDI (I, II, and III) are both involved in cell redox homeostasis. Catalase catalyses the decomposition of hydrogen peroxide to water and oxygen and its activity is used as a biomarker of oxidative stress. Therefore, it is a crucial enzyme in protecting the cell from oxidative damage by ROS and has been proposed as a biomarker and potential tool for the treatment of liver diseases like hepatitis and hepatocarcinoma [90]. Moreover, this enzyme may also control bioenergetic metabolism by regulating the activity of the Krebs cycle, respiratory chain, and ATP synthesis [90]. On the other hand, PDI acts as a converging hub for hydrogen peroxide generation pathways, including oxidases and peroxidases [91]. Moreover, it is tightly connected to oxidoreductases, mitochondria, and NADPH oxidases, the three main mechanisms of oxidant generation [91–93]. Therefore, although PDI deficiency results in health conditions [94, 95], it represents a mechanism of oxidative stress regulation. Therefore, these two seemingly opposite differential proteins, in combination with the differences in CL and ceramide lipid composition, seem to indicate that GS might be under higher levels of oxidative stress. In turn, this could be associated with dysfunctional hepatic cellular membranes and mitochondrial membranes and might explain their physiological challenges. Indeed, hepatic oxidative stress induced by diet [49, 96] or chemical exposure has been linked to decreased growth and feed efficiency in fish. Therefore, it is possible that GS fish have different nutritional needs that their current diet is unable to fulfil.

## Conclusions

Results from this study reveal ER stress as a key mechanism underlying stunted growth in seawater transfer in rainbow trout. While the drivers leading to the activation of ER stress are still unknown, proteomic and lipidomic data point towards a nutritional deficiency as an underlying driver of this phenotype. Future efforts should be directed at identifying the genetic traits of GS and re-evaluate feed formulations that can offset the nutritional deficiencies of GS.

## Methods

### Fish and rearing conditions

Post-smolt rainbow trout (*O. mykiss*) with a weight of 247.9 ± 2.21 g (mean ± SE) at seawater transfer (*n* = 306) were used in this experiment. Fish were fed *ad libidum* using a standard commercial dry diet (Skretting AS) from automatic feeders according to temperature and fish size. Fish were kept indoors in tanks equipped with LED lights in a rainbow trout facility from Lerøy Vest AS (Bjørsvik, Hordaland, Norway). Animals were kept at continuous light, natural temperature, water flow at 0.4 L/kg/min and O_2_ was above 80% in the outlet.

**Fig. 8:**
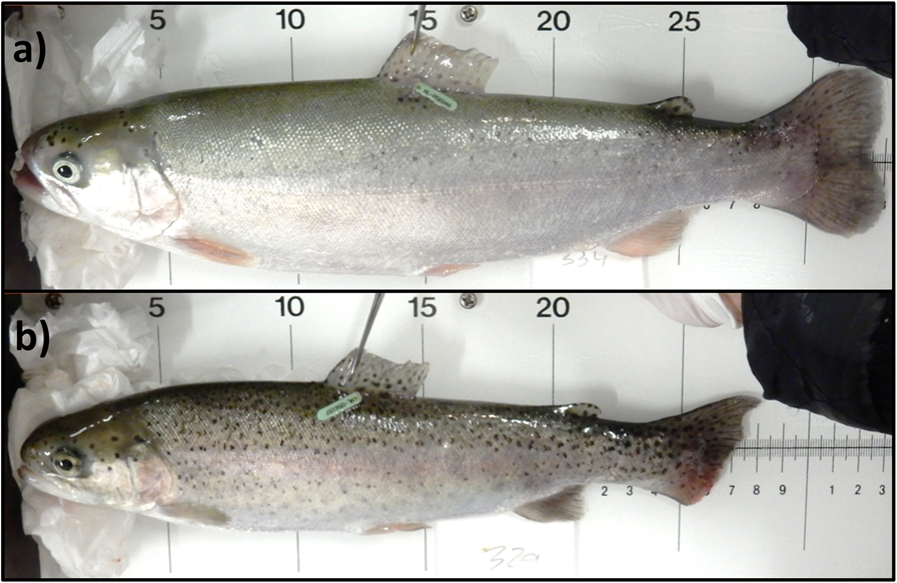
A representative specimen of the fast-growing (a) and growth-stunted (b) phenotypes in seawater-transferred rainbow trout

The present experiment was carried out on a subset of samples from *n* = 64 (*n* = 32 GS and n = 32 FG) tagged (Carlin) fish generated in Morro et al. (2019). GS presented the lowest Fulton index (< 1.25 g cm^− 3^) and standard growth rate (SGR) (< 0.65% day^− 1^) in the batch, while FG presented the highest Fulton index (> 1.45 g cm^− 3^) and SGR (> 0.65% day^− 1^). A representative specimen of each phenotype can be seen in Fig. 8.

### Sampling

Non-lethal sampling for morphometrics and blood took place on the 5th of July. Fish were transferred to seawater on the same day and lethal sampling took place 9 weeks after on the 14th of September.

For the lethal sampling, fish were quickly dip-netted out of the tanks and euthanized by a lethal overdose of isoeugenol (AQUI-S). Weight and total length were recorded. Blood was withdrawn using heparinised syringes and centrifuged at 3500×g for 10 min to obtain plasma, which was frozen at − 80 °C. The first gill arch was dissected out and preserved at − 80 °C in SEI buffer (Sucrose 250 mM, Na_2_EDTA 10 mM, Imidazole 50 mM (all Sigma-Aldrich)). Liver samples were either preserved fresh frozen at − 80 °C (for proteomic and lipidomic analysis) or preserved in RNAlater overnight at 4 °C and frozen at − 80 °C according to manufacturer guidelines (for transcription analysis).

### Gill Na^+^, K^+^–ATPase (NKA) activity

NKA activity is a measurement of osmoregulatory capacity in seawater and of the smoltification status. NKA activity was measured in gill tissue collected from 17 randomly selected fish from each group at the end-point sampling in seawater (*n* = 17).

Activity was measured following McCormick [97], which couples the hydrolysis of ATP to the enzymatic production of NAD^+^ through the involvement of the enzymes pyruvate kinase and lactate dehydrogenase, and uses the NKA inhibitor ouabain to trace the baseline. Kinetic assay readings were carried out at 340 nm for 10 min (60 cycles) at 25 °C in a Sunrise-basic (Tecan) spectrophotometer. Total amount of protein in the homogenate was analysed using a bicinchoninic acid (BCA) assay run in triplicate. NKA values were determined as the ouabain sensitive fraction of the ATP hydrolysis, expressed as μmol ADP mg protein^− 1^ h^− 1^.

### Time-resolved fluoroimmunoassay for plasma IGF-I

Circulating IGF-I levels were measured in plasma collected from 8 randomly selected fish from each group (*n* = 8). Repeated measurements took place on the same fish before seawater transfer and at the end-point sampling in seawater.

Time-resolved competitive fluoroimmunoassay (TR-FIA) was used to measure plasma IGF-I concentration [98]. Prior to the assay, plasma IGF-I was dissociated from the binding protein with acid-ethanol [99]. Specific details of the method are explained in Morro et al. [10].

### Plasma cortisol

Plasma cortisol levels were measured in plasma collected from randomly selected fish from each group, *n* = 15 in freshwater and *n* = 30 in seawater. Plasma cortisol levels of all fish sampled in freshwater (15) were measured again in seawater.

Plasma cortisol was measured using a custom enzyme-linked immunoassay (ELISA). Specific details of the method are explained in Bos et al. [100], with a volume of plasma or controls of 10 μl in this case.

### Real-time polymerase chain reaction (RT-PCR)

RT-PCR analysis was performed on liver tissue collected from 8 randomly selected fish from each group at the end-point sampling in seawater (*n* = 8).

FG and GS individuals were analysed for liver *igf1*, *igfbp1b*, *ghr1* and *ctsl* mRNA abundance as previously described in Morro et al. [10]. Briefly, 20–25 mg of liver tissue was homogenized in RLT buffer (Qiagen). Total RNA isolation was carried out using the Qia symphony RNA kit in the QIAsymphony SP automatic system following manufacturer instructions (Qiagen). Complementary DNA was reversely transcribed using 1.4 μg of total RNA using oligo (dT20) primer and the Superscript III kit (Thermo Fisher Scientific). Pipetting for both cDNA synthesis and RT-PCR was carried out using a MicrolabSTARlet Liquid Handling Workstation (Hamilton Robotics). RT-PCR was performed in a CFX-96 Real-Time PCR platform (Bio-Rad) using the following PCR conditions: 3 min at 95 °C, 34 cycles of 15 s at 95 °C and 1 min at 60 °C and a melting curve step at the end (10 s at 95 °C, 5 s at 65–95 °C with increments of 0.5 °C and 5 s at 95 °C). Samples were run in 25 μl duplicates using iTaq universal SYBR green supermix (Bio-Rad), 0.20 μM of each primer and 5 μl of 1:30 diluted cDNA. Samples with a coefficient of variation between duplicates above 1.25% were run again. The relative transcription levels of the genes were normalized following the efficiency corrected method [101] using ef1α as an endogenous reference gene [102]. Primers used in this study are summarized in Table 1.

**Table 1:** **Primers used for RT-PCR analysis.** Accession numbers of the gene sequences were obtained from GeneBank

Gene name	Primer sequence (5′ > 3′)	Accession number	Reference
*Igf1*	TGCGGAGAGAGAGGCTTTTA AGCACTCGTCCACAATACCA	M81904	[103]
*igfbp1b*	AGTTCACCAACTTCTACCTACC GACGACTCACACTGCTTGGC	AF403539	[104]
*ghr1*	CGTCCTCATCCTTCCAGTTTTA GTTCTGTGAGGTTCTGGAAAAC	AF403539	[104]
*ctsl*	CAACTACCTGCAGGCACCTA ACATGATCCCTGGTCCTTGAC	AF358668	[103]
*efα1*	CCCCTCCAGGATGTCTACAAA CACACGGCCCACGGGTACT	AF498320	[105]

### Liver proteome analysis

#### Samples

To compare the liver proteome of the FG and GS phenotypes, livers of 5 FG and 5 GS individuals were selected. The criteria for GS were low weight (< 285 g), Fulton index (< 1.21 g cm^− 3^) and SGR (< 0.5% day^− 1^) while for FG it was high weight (> 460 g), Fulton index (> 1.50 g cm^− 3^) and growth (> 0.9% day^− 1^) (Table 2).

**Table 2:** **Growth attributes of fish included in the FG and GS phenotypes**. Samples were analysed by LC-MS/MS (proteomics) and LC-MS (lipidomics)

Sample	Length (cm)	Weight (g)	Fulton index	SGR
FG	31.7 ± 0.28^a^	494.3 ± 9.97^a^	1.6 ± 0.03^a^	1.0 ± 0.08^a^
GS	26.8 ± 0.70^b^	230.2 ± 18.56^b^	1.2 ± 0.01^b^	0.1 ± 0.14^b^

Length, weight and Fulton index correspond to end-point sampling measurements. Fulton index is measured in g cm^− 3^. SGR is measured in weight gain (%) day^− 1^.

#### Liver sample preparation

Homogenization of 50 mg of liver was done in 1 ml of 0.1 M Tris-HCl pH 7.6 supplemented with 1% protease inhibitor cocktail (Roche) using a pestle motor mixer. Sodium dodecyl sulphate and dithiothreitol were added to the homogenates to a final concentration of 4% and 0.1 M, respectively. The samples were then incubated at 95 °C for 5 min and cleared by centrifugation at 16,000 xg for 10 min at room temperature.

#### TMT labelling

After measuring protein concentration by BCA (Interchim Uptima) equal amounts of protein (100 μg) were reduced, alkylated, precipitated, trypsin digested and TMT labelled following manufacturer instructions (TMT10plex™ Isobaric Label Reagent Set, ThermoFisher Scientific). Multiplexed peptide samples were cleaned-up using Hypersep SpinTip (ThermoFisher Scientific), according to manufacturer instructions. Finally, samples were dried using a vacuum drier (Savant DNA SpeedVac 110, Thermo Scientific).

#### LC MS/MS analysis of TMT

Samples were analysed with an LTQ-Orbitrap XL LC − MSn mass spectrometer (Thermo Fisher Scientific) equipped with a nanospray source and coupled to an Ultra High Pressure Liquid Chromatographer system (Waters nanoAcquity). Initially, 5 μL of sample resuspended in ultrapure water was loaded, desalted and concentrated in a BEH C18 trapping columns (Waters) with the instrument operated in positive ion mode. The peptides were then separated on a BEH C18 nanocolumn (1.7 μm, 75 μm × 250 mm, Waters) at a flow rate of 300 nL/min using an ACN/water gradient; 1% ACN for 1 min, followed by 0–62.5% ACN over 21 min, 62.5–85% ACN for 1.5 min, 85% ACN for 2 min and 1% ACN for 15 min.

MS spectra were collected using data-dependent acquisition in the m/z range 400–2000 using a precursor ion resolution of 30,000, following which individual precursor ions (top 5) were automatically fragmented using collision induced dissociation with a relative collision energy of 35%. Dynamic exclusion was enabled with a repeat count of 2, repeat duration of 30 s and exclusion duration of 180 s.

#### LC MS/MS data analysis and sequence annotation for TMT

MS data was analysed using Proteome Discoverer (ThermoFisher Scientific). Peak integration allowed for a window tolerance of 20 ppm using the ‘most confident centroid’ method. Peptide quantification was based on TMT label abundance. Only unique peptides were used for protein quantification (i.e. peptides that could be exclusively matched to a single protein in the database). Data across samples was normalized based on protein median. Only high-confidence peptides were used for quantification. Protein abundance was further normalized by dividing it in each sample by the total abundance for that protein (sum of all 10 samples). A multiple t-test followed by FDR 5% was used to compare the abundance of each detected protein in both conditions.

Peptide sequences were annotated by database search against the rainbow trout SwissProt database, which was downloaded from MASCOT and loaded into Proteome Discoverer. The initial search parameters allowed for a single trypsin missed cleavage, carbamidomethyl modification of cysteine residues, oxidation of methionine, acetylation of N-terminal peptides, a precursor mass tolerance of 10 ppm, a fragment mass tolerance of ±0.5 Da, and an FDR of 1%. Any proteins named ‘uncharacterised’ in the rainbow trout protein database were further searched by sequence homology against the Atlantic salmon (*Salmo salar*), zebrafish (*Danio rerio*), and human (*Homo sapiens*) SwissProt databases, in this order. Only homologies of E-value higher than 0.01 were accepted as valid.

Data was tested for normal distribution and homogeneity of variance assumptions using the Shapiro and Bartlett tests, respectively. Next, multiple t-test analysis and 5% FDR correction were used.

### Liver lipidome analysis

#### Individual samples

The same liver samples from FG and GS (*n* = 5/group) described in the liver proteome section were used for lipidomic analysis (Table 2).

#### Lipid extraction

Lipid extraction was carried out following the Folch method [106, 107]. Briefly, 25 mg of liver sample were homogenized in 10 ml of chloroform/methanol (2:1), incubated on ice for 1 h, with 2.5 ml of 0.88% KCl added, vortexed, incubated on ice for 5 min and centrifuged at 400 xg for 5 mins. Afterwards, the top layer was removed by aspiration and the lower layer was filtered through paper filters (No.1, Whatman). Solvent was evaporated under a stream of oxygen-free nitrogen and desiccated in vacuo overnight. Samples were stored under argon at − 20 °C.

#### LC MS/MS analysis of lipids

Lipids were analysed by LC-MS using a Thermo Orbitrap Exactive MS (Thermo Scientific), equipped with a heated electrospray ionization probe and coupled to a Thermo Accela1250U HPLC system. All samples were analysed in both positive and negative ionization modes over the m/z range 200–2000. The samples were injected into a Thermo Hypersil Gold C18 column (2.1 mm × 100 mm, 1.9 mm). Mobile phase A consisted of water containing 10 mM ammonium formate and 0.1% (v/v) formic acid. Mobile phase B consisted of 9:1 isopropanol/ACN containing 10 mM ammonium formate and 0.1% (v/v) formic acid. All solvents were LC-MS grade (Fisher Scientific). The initial conditions for analysis were 65%A/35% B. The percentage of mobile phase B was increased to 100% over 10 min and held for 7 mins before re-equilibration with the starting conditions for 4 mins.

#### LC MS/MS data analysis and lipid identification

Raw LC-MS data was processed with Progenesis QI v2.4 software (Non-linear Dynamics). Relative fold quantification was performed by the software using all ion normalization, followed by data filtering based on the ANOVA score (< 0.05), fold change (> 1.5) and ANOVA FDR (< 0.05). This was performed for data acquired in both positive and negative ionization modes. Retention and mass aligned feature data were exported for multivariate analysis (Simca-P v12.0), with OPLS-DA with parametric scaling used for data analysis [108]. An S-plot was used to identify features of interest - features with a w (1) score higher than +/− 0.04 and a p (corr) score greater than +/− 0.6 selected. These features were then identified using both the Lipid Maps and Lipidblast databases.

### General data analysis and representation

Figures were plotted using R statistical software and R package ggplot2 [109].

Differential proteins and differential lipids (both q-value < 0.05) were plotted in heatmap form showing individual sample abundance scaled by protein (i.e. by row) for liver proteins.

Unpaired t-test was used to test for differences in morphometric data, liver protein and lipid percentage, NKA activity, IGF-I abundance, cortisol abundance, and gene transcription among FG and GS groups. Data was transformed by either natural logarithm or square root to satisfy the normal distribution and homogeneity of variance assumptions, tested with the Shapiro and Bartlett tests, respectively. Similarly, paired t-test was used to test for differences between freshwater and seawater IGF-I abundance.

## Supplementary Information

**Additional File 1.** TMT proteome dataset output from Proteome discoverer on FG and GS fish liver samples.

**Additional File 2.** Lipidomic dataset containing lipid features found to distinguish FG and GS fish. Compounds found to significantly drive the separation were identified using OPLS-DA analysis (w [1] score > +/− 0.04 and a p (corr) [1] score > +/− 0.6).

**Additional File 3.** Suppl. Fig. 1. S-plot of lipidomics data between FG and GS phenotypes. The x-axis represents the contribution value that separates the experimental groups (w [1]) while the y-axis represents the reliability (pcorr [1]) . Each point represents a single m/z feature data pair. The further the data point is from the origin the greater the contribution is from this feature to differentiate among the two groups. Feature information and numerical data available in Additional File 2

## Data Availability

All data generated or analysed during this study are included in this published article [and its supplementary information files]. The mass spectrometry proteomics data generated during the current study has been deposited to the ProteomeXchange Consortium via the PRIDE partner repository with the dataset identifier PXD026897.

## Abbreviations

AWERB: Animal Welfare and Ethical Review Body
BCA: Bicinchonic acid assay
CL: Cardiolipins
CTSL: Cathepsin L
ER: Endoplasmic reticulum
ELISA: Enzyme-linked immunoassay
FG: Fast-growing
GHR1: Growth hormone receptor 1
GS: Growth-stunted
IGF-I: Insulin-like growth factor 1
IGFBP1: Insulin-like growth factor binding protein 1
LC-MS: Liquid chromatography - mass spectrometry
LC-MS/MS: Liquid chromatography - tandem mass spectrometry
NKA: Na^+^, K^+^–ATPase
NARA: Norwegian Animal Research Authority
OPLS-DA: Orthogonal partial Least-squares discriminant analysis
PC: Phosphatidylcholine
PE: Phosphatidylethanolamine
PI: Phosphatidylinositol
PS: Phostidylserine
PDI: Protein disulphide isomerases
ROS: Reactive oxygen species
RT-PCR: Real-time polymerase chain reaction
SGR: Standard growth rate
SCP-2: Sterol carrier protein 2
TR-FIA: Time-resolved competitive fluoroimmunoassay
TAGs: Triacylglycerides
UPR: Unfolded protein response
