# Supplementary figures and images for: Endoplasmic reticulum stress as a key mechanism in stunted growth of seawater rainbow trout (Oncorhynchus mykiss)
Source: BMC Genomics. 2021 Nov 16;22:824. doi: 10.1186/s12864-021-08153-5 (PMC8594166; doi:10.1186/s12864-021-08153-5)

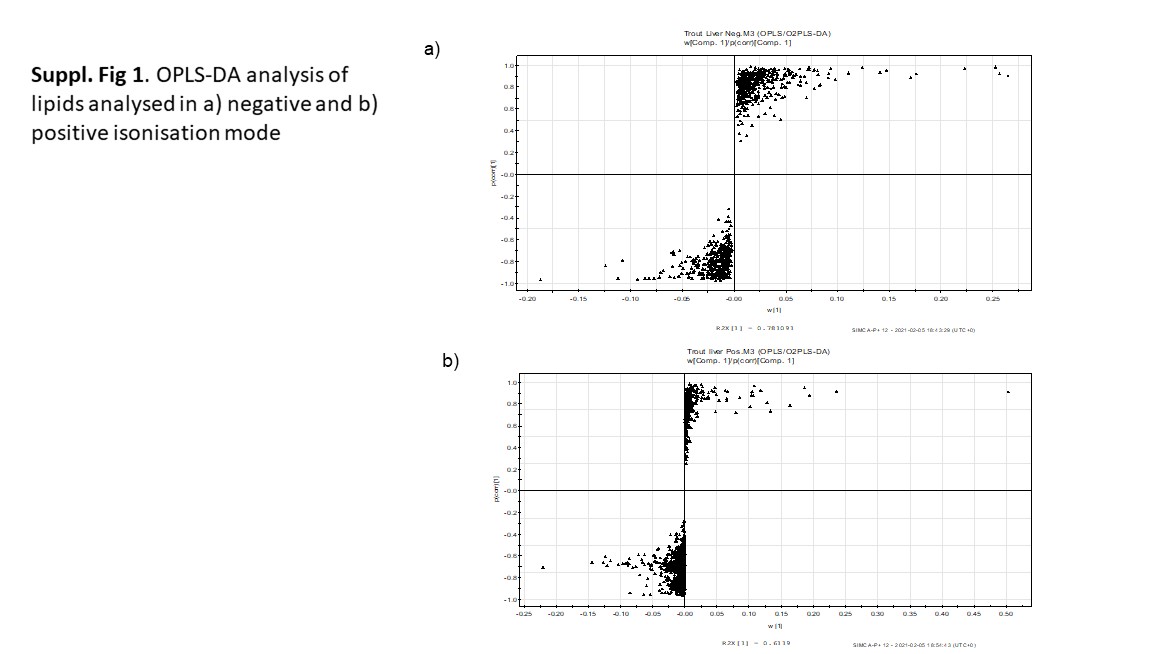

Supplement: Supplementary file 3 — Additional File 3. Suppl. Fig. 1. S-plot of lipidomics data between FG and GS phenotypes. The x-axis represents the contribution value that separates the experimental groups (w [1]) while the y-axis represents the reliability (pcorr [1]) . Each point represents a single m/z feature data pair. The further the data point is from the origin the greater the contribution is from this feature to differentiate among the two groups. Feature information and numerical data available in Additional File 2 [file 12864_2021_8153_MOESM3_ESM.jpg]
